# Evaluating the multitargeted potency of Pixuvri against cell cycle regulation proteins in cervical cancer

**DOI:** 10.3389/fonc.2025.1598883

**Published:** 2025-05-30

**Authors:** Mohammed Ali Alshehri, Hadil Alahdal, Akram Ahmed Aloqbi, Afnan Mohammed Shakoori, Ghadir Sindi, Rehab M. Bagadood, Nida Alsaffar, Mohammad Ahmad Alobaidy, Noof Abdulrahman Alrabiah, Md Khursheed, Abeer Al Tuwaijri

**Affiliations:** ^1^ Department of Clinical Laboratory Sciences, Faculty of Applied Medical Sciences, Najran University, Najran, Saudi Arabia; ^2^ Department of Biology, College of Science, Princess Nourah bint Abdulrahman University, Riyadh, Saudi Arabia; ^3^ Department of Biological Science, Faculty of Science, University of Jeddah, Jeddah, Saudi Arabia; ^4^ Department of Clinical Laboratory Sciences, Faculty of Applied Medical Sciences, Umm Al-Qura University, Makkah, Saudi Arabia; ^5^ Department of Medical Laboratory Sciences, Mohammed Al-Mana College for Medical Sciences, Dammam, Saudi Arabia; ^6^ Department of Anatomy, Faculty of Medicine, Umm Al-Qura University, Makkah, Saudi Arabia; ^7^ Department of Biological Sciences, College of Science, King Faisal University, Al Ahsa, Saudi Arabia; ^8^ Department of Molecular Physiology, Mohammed Bin Rashid University, (MBRU), Dubai, United Arab Emirates; ^9^ Medical Genomics Research Department, King Abdullah International Medical Research Center (KAIMRC), Ministry of National Guard Health Affairs, Riyadh, Saudi Arabia; ^10^ Department of Clinical Laboratory Sciences, College of Applied Medical Sciences, King Saud Bin Abdulaziz University for Health Sciences, Riyadh, Saudi Arabia

**Keywords:** cervical cancer, multitargeted drugs, drug resistance, Pixuvri, WaterMap

## Abstract

Cervical cancer, primarily caused by persistent infection with high-risk human papillomavirus strains, leads to abnormal cell growth in the cervix. Globally, it accounts for over 600,000 new cases and 340,000 deaths annually, with the highest burden in low- and middle-income countries due to limited screening and vaccination. Early detection is challenging as initial stages are asymptomatic, while advanced cases are challenging to treat. Current options, including surgery, radiotherapy, and chemotherapy, face issues like toxicity, limited efficacy, recurrence, and drug resistance caused by tumor heterogeneity and adaptive mechanisms. Multitargeted drug design offers a solution by modulating multiple cancer pathways, enhancing efficacy, minimizing resistance, and reducing side effects. In this study, we screened Selleckchem approved library against cervical cancer proteins that regulate the cell cycle, particularly during mitosis and cell division (PDBIDs: 2VFX, 2WVI, 3KND, 4N14) using HTVS, SP, and XP docking followed by MMGBSA post-processing. Pixuvri (Pixantrone Maleate) emerged as the top candidate with docking scores of -5.234 to -9.218 kcal/mol and MMGBSA scores of -39.22 to -53.87 kcal/mol. Pixuvri is approved for non-Hodgkin lymphoma and exhibits minimal cardiotoxicity compared to anthracyclines. Interaction fingerprints highlighted key residues (4GLN, 4GLU, 3TRP), while pharmacokinetics, DFT computations, and WaterMap hydration site analysis supported its potential. Molecular dynamics (100 ns, NVT ensemble at 300K) validated stability by deviation and fluctuation studies and found many interactions to stabilize the complex, with binding free energy computations confirming its affinity. While the results support Pixuvri’s repurposing for cervical cancer, experimental validation is essential for clinical application.

## 1 Introduction

Cervical cancer is a major global health issue, especially for women in low- and middle-income countries, that originates in the cervix and is mainly caused by persistent infection with high-risk HPV strains (1–3). Despite advancements in preventative measures such as HPV vaccination and cervical screening programs, cervical cancer remains a major contributor to cancer-related morbidity and mortality worldwide. According to recent statistics, over 600,000 new cases of cervical cancer are diagnosed annually, and more than 340,000 women lose their lives to this disease, underscoring the urgent need for improved therapeutic strategies (4, 5). Cervical cancer disproportionately affects women in resource-limited settings, where access to screening and vaccination programs is restricted. The disease often progresses to advanced stages in such regions before diagnosis, making treating it more challenging. The WHO emphasizes that cervical cancer is one of the most preventable and treatable forms of cancer if detected early (6, 7). However, the lack of awareness, infrastructure, and healthcare resources perpetuates high mortality rates in underserved populations. The global disparity in cervical cancer outcomes highlights the need for comprehensive approaches that integrate prevention, early detection, and innovative treatment strategies (8). The primary cause of cervical cancer is persistent infection with oncogenic HPV types, particularly HPV-16 and HPV-18, which together account for approximately 70% of all cases. HPV is a sexually transmitted virus, and most infections are transient, cleared by the immune system within a few months to years. However, in a subset of women, persistent HPV infection leads to the integration of viral DNA into the host genome (9, 10). This integration disrupts cellular regulatory pathways by overexpressing viral oncoproteins E6 and E7, which inactivate tumor suppressor proteins p53 and retinoblastoma (Rb), respectively. The resulting genomic instability and uncontrolled cell proliferation are hallmarks of cervical carcinogenesis (11). Tumor cells frequently develop resistance to conventional chemotherapeutic agents like cisplatin, limiting treatment efficacy. Resistance mechanisms include enhanced DNA repair, altered drug metabolism, and evasion of apoptosis (12).

Cervical cancer is driven by dysregulation in critical molecular pathways that govern cell division, mitotic progression, and chromosomal stability. In this study, we have taken four crucial proteins that play pivotal roles in these processes and have been selected for a multitargeted docking study to identify potential therapeutic inhibitors (5, 13–17). The symmetric Mad2 dimer (PDB ID: 2VFX) is a key component of the spindle assembly checkpoint (SAC), ensuring accurate chromosome segregation during mitosis. In cervical cancer, Mad2 dysregulation causes chromosomal instability and aneuploidy, driving tumor progression. Drugs targeting Mad2 dimerization or its interactions with other SAC components could restore checkpoint function and inhibit tumor growth (18). The N-terminal domain of BubR1 (PDB ID: 2WVI) is crucial in the SAC, functioning as a mitotic checkpoint kinase to prevent early anaphase onset. In cervical cancer, BubR1 overexpression or mutations lead to abnormal mitosis and genomic instability. Targeting BubR1’s N-terminal domain with multitargeted inhibitors can disrupt its interactions with other SAC proteins, improving checkpoint accuracy and reducing tumor cell viability (19). The TPX2-importin-alpha complex (PDB ID: 3KND) is vital for spindle assembly and mitotic progression. In cervical cancer, TPX2 is often overexpressed, causing abnormal spindle formation and unchecked cell division. The complex regulates TPX2’s localization and activity. Multitargeted inhibitors that disrupt this complex can impair spindle assembly, inducing mitotic arrest and apoptosis in cancer cells (20). Cdc20 (PDB ID: 4N14) activates the anaphase-promoting complex/cyclosome (APC/C), driving the transition from metaphase to anaphase. In cervical cancer, Cdc20 overactivation causes premature chromosomal segregation and genomic instability. The Cdc20 complex regulates APC/C activation. Multitargeted drugs that block this interaction can inhibit APC/C activity, inducing mitotic arrest and preventing tumor cell proliferation (21). By simultaneously targeting these proteins, multitargeted drugs can disrupt the interconnected pathways driving cervical cancer progression. This strategy may overcome tumor heterogeneity, reduce resistance, and improve therapeutic efficacy. Computational techniques like molecular docking, molecular dynamics simulations, and binding free energy calculations will be key in identifying and optimizing compounds that effectively inhibit these targets (22–24).

In recent years, multitargeted drug design has emerged as a promising strategy for addressing the complexities of cervical cancer treatment. Unlike traditional single-target therapies, multitargeted drugs are designed to modulate multiple molecular pathways simultaneously. This approach is particularly advantageous for a disease like cervical cancer, where multiple genetic and epigenetic alterations drive tumor progression and resistance. Multitargeted drugs can reduce the likelihood of resistance development by targeting multiple pathways involved in tumor survival and proliferation (5, 14, 25). For instance, inhibiting both angiogenesis and cell cycle progression simultaneously can disrupt complementary survival mechanisms employed by cancer cells. Combining multiple targets within a single drug reduces the need for combination therapies, which often exacerbate toxicity. Furthermore, multitargeted drugs can be designed to act on diverse molecular subtypes, making them effective against heterogeneous tumor populations. While the concept of multitargeted drug design holds great promise, its implementation comes with significant challenges (16, 17, 22). Identifying and validating multiple relevant targets requires extensive research and computational modelling. Broad-spectrum activity can sometimes result in unintended interactions with healthy tissues, leading to adverse effects. Ensuring a multitargeted drug achieves effective concentrations at all intended targets while minimizing toxicity is complex (26). Computational methods are pivotal in accelerating the discovery and optimization of multitargeted medicines. Techniques such as molecular docking predict the binding affinity of potential drug candidates to multiple targets, enabling the identification of compounds with broad-spectrum activity. Density Functional Theory (DFT) calculations provide insights into the electronic properties of molecules, aiding in the optimization of drug-target interactions (25, 27). Molecular dynamics (MD) simulations provide a dynamic perspective of drug-target interactions, tracking conformational changes and stability over time. WaterMap analysis identifies thermodynamically unfavorable water molecules at the binding site, which, when displaced, can enhance binding affinity. Binding free energy calculations assess the thermodynamic favorability of drug-target interactions, helping select high-affinity drug development candidates (28, 29).

In this study, we performed a multitargeted docking study of Selleckchem approved library with multiple cervical cancer proteins followed by molecular interaction fingerprints. The study was extended by DFT and pharmacokinetics on identified molecules. Further, we also performed the 5ns WaterMap and MD simulation for 100ns, followed by the binding free energy computations to provide better evidence that the identified drug can be used as a multitargeted candidate against cervical cancer.

## 2 Methods

**Figure 1:**
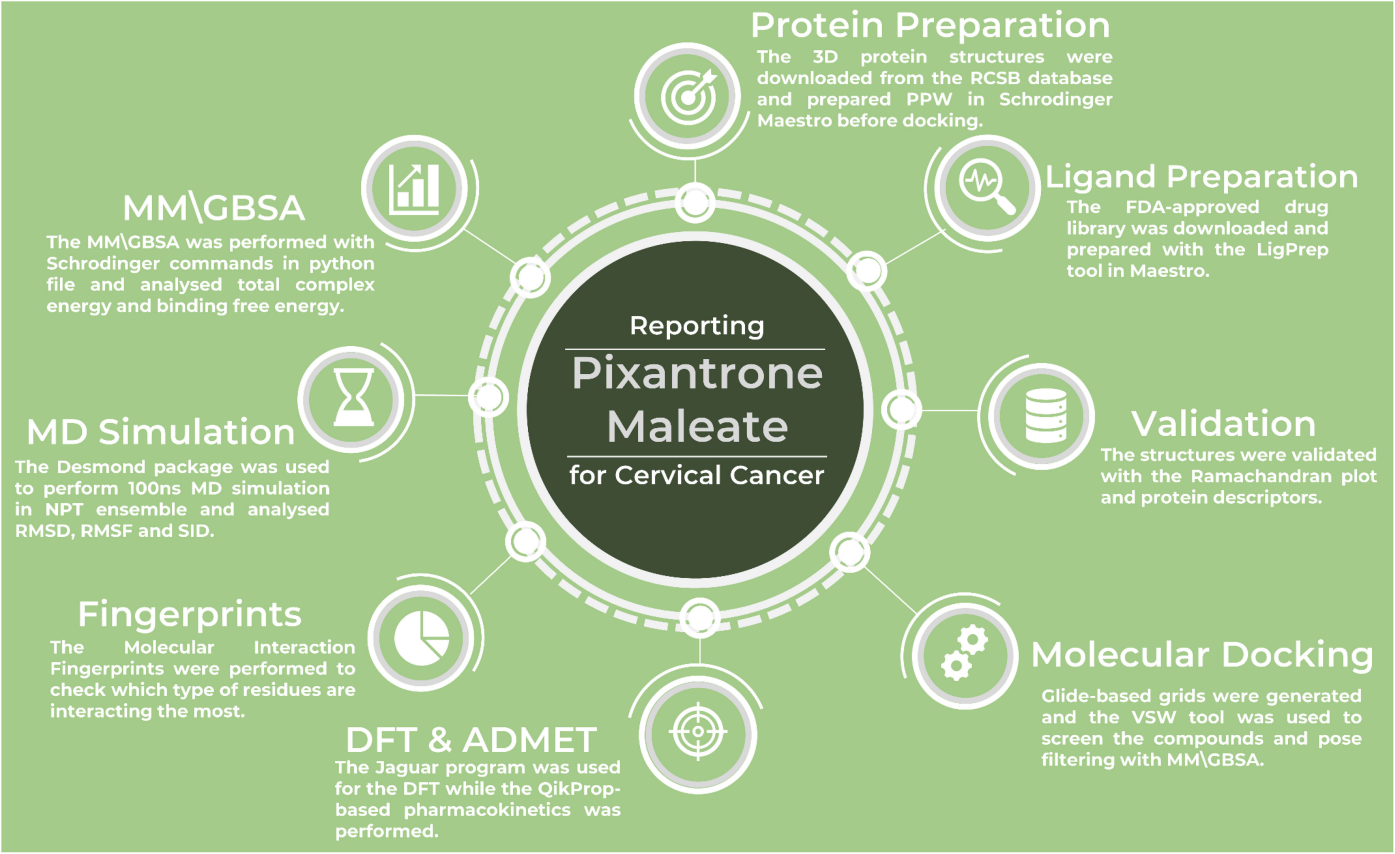
The workflow of the complete study to identify and validate the multitargeted drug candidate against cervical cancer proteins.

In this study, to identify and validate the multitargeted drug against cervical cancer, we performed several studies, as shown in **Figure 1**, and the detailed methods are as follows-

### 2.1 Ligand and protein data collection and preparation

The FDA-approved ligand library was sourced from Selleckchem (https://www.selleckchem.com/) and processed using Schrödinger Maestro’s LigPrep tool. Compounds with more than 500 atoms were removed, and potential forms of the compounds were created based on a target pH of 7 ± 2 using the Epik module. The compounds were then desalted, and tautomers were generated. Stereoisomers were calculated while keeping the specified chiralities, with up to 32 versions of each compound produced (30–32). The processed library was saved in SDF format and utilized for docking studies. Protein data collection involves obtaining structural information through X-ray crystallography, NMR spectroscopy, or cryo-electron microscopy techniques. This data provides insights into the 3D structure of the protein, which is crucial for understanding its function and interaction with ligands. Protein preparation typically includes removing water molecules, adding missing atoms or residues, optimizing the structure, and assigning appropriate force field parameters. These steps ensure that the protein model is ready for docking, molecular dynamics simulations, or other computational analyses (10, 30, 33). Proper preparation of protein structures is essential for addressing issues like incomplete side chains or unfavorable geometries, ensuring the model is both biologically accurate and computationally efficient. This preparation improves the reliability of docking results and other computational studies, which is crucial for drug discovery and design. The protein structures used in this study, including the BubR1 protein (PDB ID: 2WVI), Cell Division Cycle Protein 20 Homolog (Cdc20, PDB ID: 4N14), MAD2 protein (PDB ID: 2VFX), TPX2 protein (PDB ID: 3KND), were obtained from the RCSB PDB database (https://www.rcsb.org/) and then these structures were then prepared using Schrödinger’s Maestro software and its Protein Preparation Workflow (PPW) (18–21, 31, 34, 35). The downloaded protein structures contained the following components: Chain A, solvents and other metals/ions in 2WVI; Chain A, ligands and solvents in 4N14; Chain A to L, solvents, and ligands in 2VFX; and Chains A, ligands, and solvents in 3KND. All four protein structures were imported into the project table of the PPW in Schrödinger. The preparation process involved capping the termini, filling in missing side chains, assigning bond orders, creating disulfide bonds, and setting zero bond orders for metals. Missing loops were rebuilt using Prime, and heteroatom states were generated using Epik at a pH of 7.4 ± 2 (30, 31, 36). The protein structures were assessed to ensure proper water orientation, and the hydrogen positions of altered species were minimized. pH adjustments were made using PROPKA (37). Following this, energy minimization was performed on all atoms, with a maximum deviation of 0.30 Å, using the OPLS4 force field. To further refine the structures, water molecules located more than 5 Å from the ligand were removed (31, 37, 38). After preparation, we kept only Chain A in all cases to keep the clean structures of the protein for generating the grids on it for the docking studies.

### 2.2 Receptor grid generation and molecular docking studies

Receptor grid generation is key in molecular docking, creating a 3D grid around the protein’s binding site to define ligand interaction regions. It is based on electrostatic and steric properties, improving docking accuracy and efficiency. In this study, grids were created for all protein structures to perform blind docking and identify optimal multitarget candidates. Using Schrödinger’s Receptor Grid Generation tool in Maestro, we set a scaling factor of 1 and a partial charge cutoff of 0.25. The grid box was configured to cover all residues at the site, and the grid was cantered to include the entire protein. The “Dock ligands with length” parameter was adjusted to ensure a proper fit. The resulting grid was saved in a zip format for the multitargeted docking process (31, 39). Molecular docking studies simulate the binding of a ligand to a receptor, predicting the most favorable binding conformation and affinity. The process involves the ligand fitting into the receptor’s binding site, considering factors such as shape complementarity, electrostatic interactions, and hydrogen bonding. These simulations provide insights into the molecular interactions between the ligand and receptor, aiding drug discovery and designing more effective therapeutic agents. The results are typically evaluated using scoring functions to determine the best binding poses. In this study, we used Schrödinger’s Virtual Screening Workflow (VSW) in Maestro to explore the prepared ligand library, ensuring that each unique compound had distinct properties to avoid screening duplicates and save time (31, 40). For compound filtering, we utilized QikProp to generate the pharmacokinetic properties of the ligands and applied Lipinski’s rule to refine the selection further. After preparing the ligands, the grid was accessed individually through the receptor tab (31, 41–43). We considered the enhanced planarity of conjugated pi-groups in the docking tab and applied Epik state penalties to improve the docking process (30). The scaling factor was set to 0.80, and the partial charge cutoff was set to 0.15. We performed docking using three strategies: high throughput virtual screening (HTVS), standard precision (SP), and extra precision (XP). After docking, molecular mechanics with generalized Born and surface area (MM/GBSA) calculations were applied to each pose. All 100 filtered ligands were initially docked using HTVS, with the top 10% advancing to SP and the top 50% of SP results moving on to XP. Four poses per compound were generated in XP, and all XP results were passed through MM/GBSA for further analysis. The results were exported in CSV format for detailed analysis, helping us identify the best compound based on docking scores.

### 2.3 Interaction fingerprints and pharmacokinetics studies

Interaction fingerprints are computational representations that capture key binding interactions like hydrogen bonds, hydrophobic, electrostatic, and van der Waals forces in binary or numerical formats. By comparing these fingerprints, researchers can identify common interaction patterns, aiding in understanding binding mechanisms and optimizing drug design for more effective compounds (14, 15, 25). Molecular Interaction Fingerprints (MIFs) are created by mapping interactions at each residue or region of the protein’s binding site during docking or simulation studies. These fingerprints allow for easy comparison of binding patterns across different ligands or targets, supporting structure-activity relationship (SAR) studies, virtual screening, and multitarget optimization. MIFs are especially helpful in identifying common interaction profiles among ligands, which can aid in designing compounds with better specificity and effectiveness. To generate MIFs, we used the Interaction Fingerprint panel in Schrödinger Maestro (31). We selected all four protein-ligand complexes, chose the receptor-ligand complex option, and aligned the data with PDBID-2WVI as the reference. The fingerprints were generated and analyzed using the resulting matrix. We included all interactions and visualized the main plot with color coding to distinguish the protein’s N- to C-terminal regions. Only interacting residues were considered for analysis, and the count of residue-ligand interactions was reviewed to pinpoint key binding features. Pharmacokinetics (PK) studies focus on understanding how a drug behaves in the body over time, including its absorption, distribution, metabolism, and excretion (ADME). These studies help predict a drug’s bioavailability, half-life, and potential side effects. Computational PK modelling uses the physicochemical properties of the drug, such as molecular weight, lipophilicity, and solubility, to estimate these parameters. In drug discovery, PK studies guide the optimization of drug candidates by identifying properties that promote better absorption, effective distribution, and minimal toxicity, ensuring therapeutic efficacy. For the analysis of the pharmacokinetics of the identified drug candidate, we used the QikProp panel in Schrödinger’s Maestro, applied Lipinski’s rule to ensure proper evaluation and analyzed various descriptors while comparing them with the standard values (31, 41–44).

### 2.4 Density functional theory computations

Density Functional Theory (DFT) is a quantum mechanical method that studies molecular electronic structures by focusing on electron density. In drug design, DFT optimizes ligands for better binding affinity and stability, predicting properties like the HOMO-LUMO gap and dipole moment. These insights help design effective, targeted drugs by understanding molecular behavior and interactions (45). For this study, DFT calculations were performed using the Optimization panel in Schrödinger’s Maestro software, which relies on Jaguar for the computational work. We used the B3LYP-D3 method and the 6-31G** basis set to model the electronic structure and optimize the geometry of the molecules. This approach allowed us to accurately predict the studied compounds’ electronic properties and energy characteristics (31, 46–48). The DFT calculations included automatic treatment of the SCF spin and a quick level of atomic overlap (31, 49, 50). The calculations were set to run for a maximum of 48 iterations, with an energy change threshold of 5e-05 Hartree and 100 steps for optimization using default convergence criteria. During the process, various dynamics were calculated, such as molecular orbitals, HOMO, and LUMO. We used the PBF solvent model to simulate water solvents, and the results were saved for further analysis, which was done using the QM-convergence monitor panel in Maestro (31).

### 2.5 WaterMap computations

WaterMap is a tool that analyses the role of water molecules in protein-ligand binding. It predicts water molecule locations within the binding site and their impact on ligand stability. By assessing water displacement energetics, WaterMap helps optimize drug design by identifying unfavorable water molecules that can be displaced to enhance binding affinity and specificity (31, 51). WaterMap identifies hydration hot spots where water molecules stabilize protein-ligand interactions, guiding ligand optimization in structure-based drug design (SBDD). It helps replace unfavorable waters and incorporate essential ones, improving binding affinity and selectivity. This tool is vital for lead optimization, revealing the role of water in binding at the molecular level (29, 51). For the WaterMap calculations, we used the WaterMap-perform calculations panel in Schrödinger’s Maestro. We selected the docked ligand in the protein-ligand complex and focused on analyzing water molecules within 10 Å of the ligand (31, 51, 52). The simulation was set up by truncating the protein, using the OPLS4 force field, and treating existing water molecules as solvents. The simulation time was kept to 5 ns to avoid generating trajectory files (31, 38). The results were analyzed using the WaterMap-Examine Results panel in Maestro, where we examined water molecules’ thermodynamics and hydration sites, including enthalpy, entropy, free energy, and overlap factors (31, 51, 52).

### 2.6 Molecular dynamics simulation and binding free energy computations

MD simulations model the time-dependent behavior of molecules by solving Newton’s equations of motion. They provide insights into protein-ligand interactions, protein folding, and conformational changes over time. MD helps assess biomolecule stability, flexibility, and function in a physiological environment, which is crucial for drug-binding mechanisms and optimizing drug candidates (26). The MD simulations for all four protein-ligand (PL) complexes were carried out using the Desmond package (https://www.deshawresearch.com/resources.html), integrated into Schrödinger’s Maestro software. The simulations followed a three-step process to ensure accuracy and reliability (31, 53). The first step involved preparing the system file using the System Builder in Maestro. We chose the TIP3P water model for solvation, set up a buffer box of 10 × 10 × 10 Å, and minimized the volume to ensure the box was appropriately sized. The box boundary was checked to confirm that it fully encompassed the protein-ligand complex, ensuring proper system configuration for the MD simulation (31, 54). We excluded ion and salt placements within 20 Å of the protein-ligand complex and then added ions to neutralize the system: 7 Na+ and 5 Cl- ions for the 2WVI complex, 2 Cl- and 9 Na+ ions for the 2B9R complex, and the corresponding ions for the 3VHX and 3KND complexes in combination with Droxidopa. The OPLS4 force field was applied to facilitate the MD simulation, and the setup was finalized for the run. This ionization process ensures the systems are electrically neutral and properly solvated for accurate simulations (31, 38). In the second step, we used the Molecular Dynamics panel in Schrödinger Maestro for the production run. After loading the prepared protein-ligand complex, the simulation was set for 100 ns with a recording interval of 100 ps, resulting in 1000 frames for each condition. The NPT ensemble was selected, with the system maintained at 300K and 1.01 atm pressure. The system was also relaxed before the production run to allow for equilibration, ensuring stable and realistic conditions for the simulation (31, 55). The third step is the analysis phase, where we assessed deviations, fluctuations, and intermolecular interactions within the complexes. We focused on how the protein-ligand complex changed over time during this phase. We mainly looked at key dynamic properties like deviation, fluctuation, and ligand and protein interactions. This analysis helped us evaluate the complex’s stability and the consistency of binding interactions. The results were then plotted for better visualization, helping us understand how the ligand influenced the protein structure throughout the simulation.

Binding free energy computations estimate the strength and stability of ligand-receptor interactions, which are crucial for drug design. Methods like MM-PBSA, Thermodynamic Integration (TI), and Free Energy Perturbation (FEP) are used to predict binding affinity. Accurate predictions optimize drug candidates by identifying high-affinity and low-toxicity molecules, improving drug development (28, 47). It offers the benefit of dynamic analysis and precise ranking, making it widely used for validating docking results, optimizing lead compounds, and understanding the molecular basis of ligand binding and protein conformational changes. The Desmond-based MD simulation (31, 53) produced the trajectory for the complexes, which was then used for MM/GBSA calculations. The analysis included various energy components, such as the total complex energy and binding free energy, which were processed using the provided bash commands-

$SCHRODINGER/run thermal_mmgbsa.py desmond_NAME-out.cms

## 3 Results

### 3.1 Analysis of prepared protein descriptors and their structure validation

**Figure 2:**
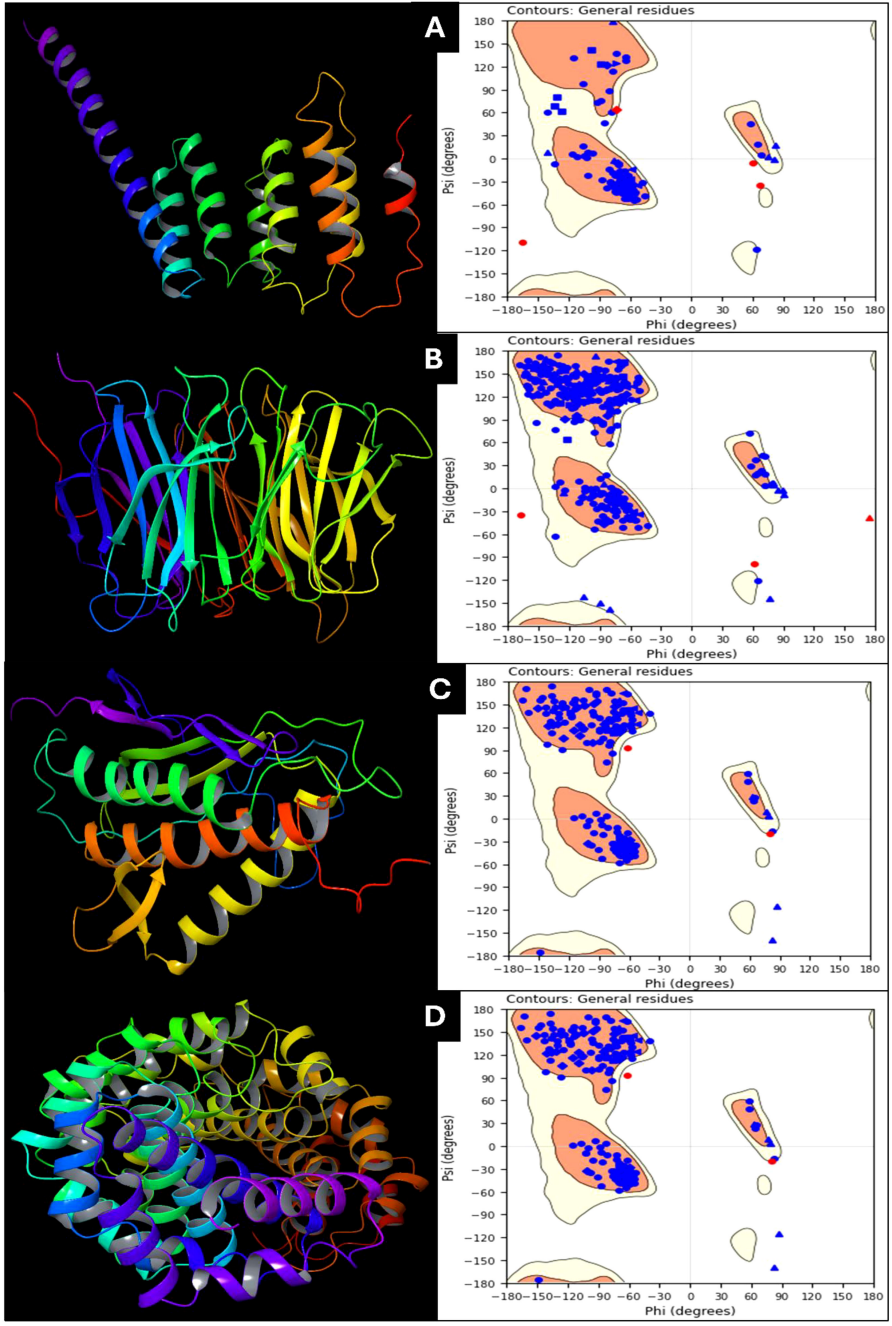
Prepared 3D structure of proteins and Ramachandran Plot for **(A)** 2VFX, **(B)** 4N14, **(C)** 2WVI, and **(D)** 3KND.

The protein preparation results for the selected PDBIDs provide insights into these crucial targets’ structural stability and interaction dynamics in cervical cancer. Among the studied proteins, the symmetric Mad2 dimer (PDB ID: 2VFX) exhibits the most significant stability, with a total energy of -15905.9 kcal/mol. This stability is attributed to strong Lennard-Jones interactions (-25766.4 kcal/mol) and electrostatic interactions (-17543.1 kcal/mol), which reflect robust van der Waals forces and favorable electrostatic binding. The high bond stretch energy (1170.01 kcal/mol) and torsion energy (4992.15 kcal/mol) further indicate the structural complexity and rigidity of the dimer. The Cdc20 and apcin complex (PDB ID: 4N14) demonstrates moderate stability, with a total energy of -1946.68 kcal/mol. Its bond stretch energy (130.384 kcal/mol) and angle bending energy (641.786 kcal/mol) reveal a well-distributed structural stress profile. Torsion energy (495.367 kcal/mol) suggests some degree of rotational flexibility, while Lennard-Jones energy (-3223.28 kcal/mol) and electrostatic energy (-1895.36 kcal/mol) highlight stable non-covalent interactions within the complex. The N-terminal domain of BubR1 (PDB ID: 2WVI) has the lowest total energy (-855.485 kcal/mol), indicating reduced inherent stability compared to the other proteins. Its bond stretch energy (80.6029 kcal/mol) and angle bending energy (355.754 kcal/mol) suggest minimal structural constraints. Lower Lennard-Jones energy (-1657.76 kcal/mol) and electrostatic energy (-1023.09 kcal/mol) reflect modest stabilizing forces, which may correspond to fewer interaction hotspots in this domain. The TPX2-importin-alpha complex (PDB ID: 3KND) displays considerable stability, with a total energy of -2891.6 kcal/mol. Bond stretch energy (220.651 kcal/mol) and angle bending energy (1107.62 kcal/mol) indicate moderate structural rigidity. Torsion energy (697.125 kcal/mol) highlights flexibility within the complex, which is essential for biological function. Lennard-Jones energy (-4665.06 kcal/mol) and electrostatic energy (-3200.14 kcal/mol) further underscore the importance of non-covalent interactions in maintaining stability. Comparatively, the symmetric Mad2 dimer exhibits the highest stability due to its strong non-covalent interactions and structural rigidity, followed by the TPX2-importin-alpha complex and the Cdc20-apcin complex. The BubR1 domain shows relatively lower stability, reflecting fewer structural constraints. These findings underscore these proteins’ structural variation and interaction dynamics, pivotal in cervical cancer progression. Further, in **Figure 2**, we have shown the prepared protein structures and their Ramachandran plots for better understanding.

### 3.2 Multitargeted interaction assessment

**Figure 3:**
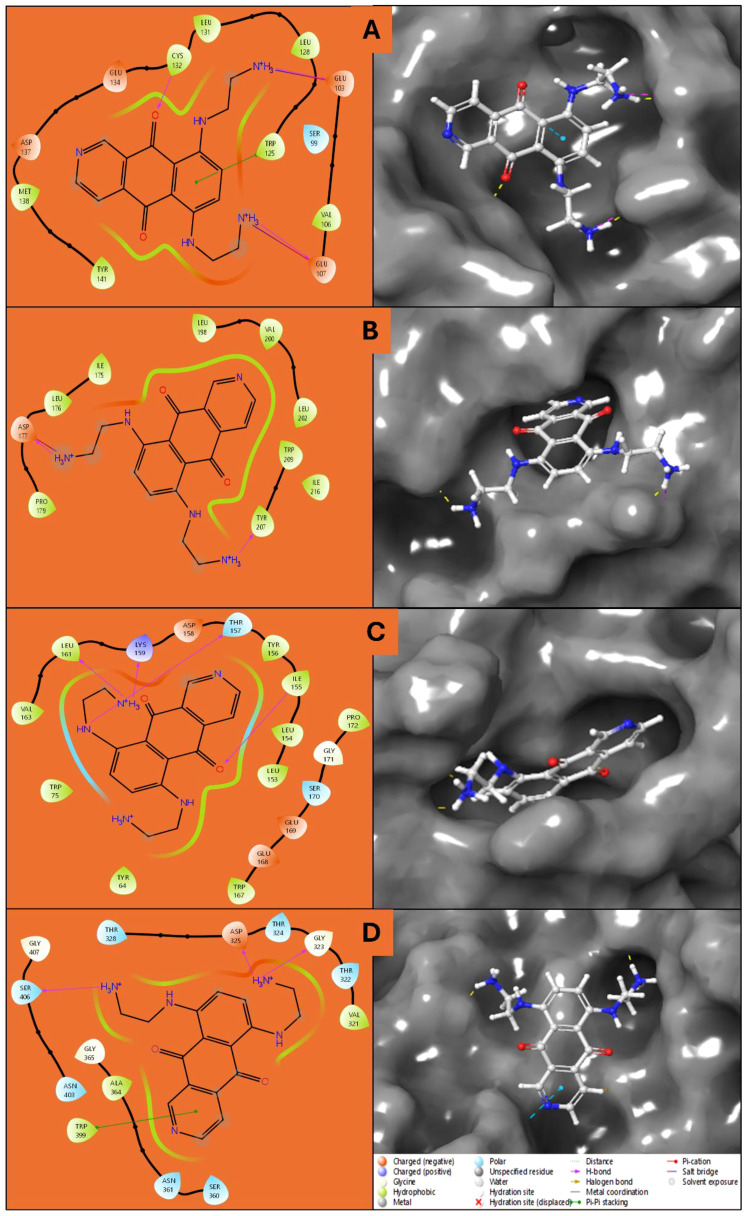
Ligand Interaction Diagram of docked poses in 2D for showing the interacting residues and bond types and 3D for fit in the pocket for **(A)** 2VFX, **(B)** 4N14, **(C)** 2WVI, and **(D)** 3KND.

The docking results of Pixuvri with key cervical cancer target proteins provide valuable insights into their interaction profiles, including docking scores, MMGBSA binding energies, and interaction details. The docking score for the BubR1 protein (PDB ID: 2WVI) was -8.92, and the MMGBSA binding energy was -44.22 kcal/mol. The ligand Pixuvri formed three hydrogen bonds involving the GLU103 and GLU107 residues with two N+H3 atoms and the CYS132 residue with the O atom. A π-π stacking interaction was also observed with the TRP125 residue along the benzene ring. Two salt bridges were also formed, involving GLU103 and GLU107 residues along two N+H3 atoms of Pixuvri. The complex energy was calculated as -7444.461 kcal/mol, with ligand efficiency (sa) of -2.764 and ligand efficiency (ln) of -10.583. These findings highlight the potential of Pixuvri to interfere with the mitotic checkpoint regulation mediated by BubR1, which is critical for proper cell cycle progression in cervical cancer cells. The Cell Division Cycle Protein 20 Homolog (Cdc20, PDB ID: 4N14) demonstrated a docking score of -5.23 and an MMGBSA binding energy of -45.40 kcal/mol. Hydrogen bond interactions were identified with the ASP177 and TYR207 residues, involving two N+H3 atoms of Pixuvri. A salt bridge was formed with ASP177 through the N+H3 atom of the ligand. The complex energy was -12622.589 kcal/mol, with a ligand efficiency (sa) of -2.837 and ligand efficiency (ln) of -10.866. This interaction suggests that Pixuvri may inhibit the activation of the anaphase-promoting complex (APC) by targeting Cdc20, thereby disrupting cell division and potentially inducing apoptosis in cancer cells. The docking score for the MAD2 protein (PDB ID: 2VFX) was -9.21, and the MMGBSA binding energy was -53.87 kcal/mol. Pixuvri formed four hydrogen bonds with LYS159 and LEU161 residues via the N+H3 atom, THR157 via the NH atom, and ILE155 via the O atom. The complex energy was -8295.563 kcal/mol, with ligand efficiency (sa) of -3.367 and ligand efficiency (ln) of -12.894. MAD2 plays a vital role in spindle assembly checkpoint signaling, and its interaction with Pixuvri suggests potential inhibition of this checkpoint, leading to mitotic arrest and subsequent cancer cell death. The TPX2 protein (PDB ID: 3KND) exhibited a docking score of -5.36 and an MMGBSA binding energy of -39.22 kcal/mol. Hydrogen bonds were formed with GLY323, ASP325, and SER406 via two N+H3 atoms of Pixuvri. A π-π stacking interaction was observed with the TRP399 residue along the benzene ring of the ligand. The complex energy for this interaction was -17333.134 kcal/mol, with ligand efficiency (sa) of -2.451 and ligand efficiency (ln) of -9.388. TPX2 is crucial for spindle assembly and microtubule stabilization during mitosis, and its interaction with Pixuvri underscores the ligand’s ability to interfere with these processes, contributing to its antimitotic effects. These results indicate strong binding affinities and favorable interaction energies for Pixuvri across the selected proteins, highlighting its potential as a multitargeted therapeutic agent in cervical cancer. The multitarget approach can overcome the limitations of single-target therapies, such as drug resistance and heterogeneous tumor profiles. By inhibiting proteins involved in distinct yet interconnected pathways, Pixuvri may exert a synergistic effect, enhancing therapeutic efficacy and reducing the likelihood of cancer recurrence. Pixuvri exhibits varying affinities and interaction profiles across key cervical cancer targets, suggesting its potential for a multitargeted approach. The docking results show strong interactions with BubR1, Cdc20, MAD2, and TPX2, which are crucial for cell cycle regulation. Among these, Pixuvri demonstrates the highest docking score and binding energy with MAD2 (-9.21 docking score and -53.87 kcal/mol MMGBSA), indicating a more substantial inhibitory potential at the spindle assembly checkpoint. However, Pixuvri also forms stable interactions with BubR1 (-8.92 docking score and -44.22 kcal/mol MMGBSA) and Cdc20 (-5.23 docking score and -45.40 kcal/mol MMGBSA), both essential for cell division. While MAD2 shows the most significant binding affinity, Pixuvri’s ability to target multiple proteins involved in different stages of mitotic regulation suggests a broader therapeutic advantage. The combined inhibition of these proteins could enhance efficacy and minimize resistance, making Pixuvri a promising multitargeted drug candidate for cervical cancer treatment. Further, **Figure 3** has 3D and 2D representations of docked poses, and **Table 1** shows various scores produced during the docking studies.

**Table 1:** Docking, MM\GBSA and other scores were produced during the multitargeted docking studies of Pixuvri’s in complex with four PDBIDs.

PDB id	State penalty	Docking score	XP GScore	XP HBond
4XZL	0.0004	-8.925	-8.926	-1.114
3IUC	0.0004	-5.234	-5.234	-0.9
6G77	0.0004	-9.218	-9.219	-1.005
4B3Z	0.0004	-5.365	-5.366	-1.6
PDB id	MMGBSA dG Bind	Complex energy	Ligand efficiency sa	Ligand efficiency ln
4XZL	-44.22	-7444.461	-2.764	-10.583
3IUC	-45.4	-12622.589	-2.837	-10.866
6G77	-53.87	-8295.563	-3.367	-12.894
4B3Z	-39.22	-17333.134	-2.451	-9.388

### 3.3 Interaction fingerprints and pharmacokinetics assessment

**Figure 4:**
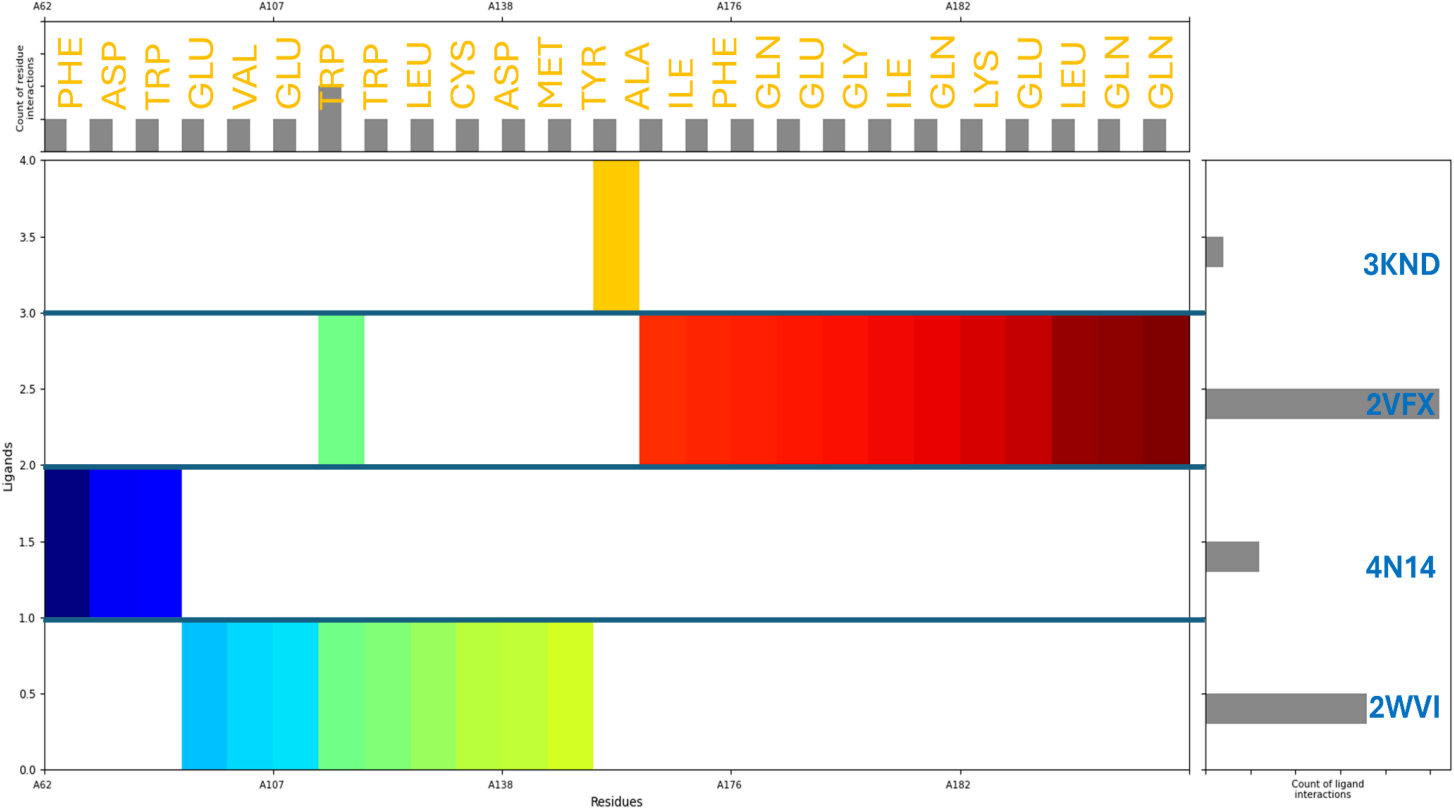
Molecular Interaction Fingerprints of Pixuvri in complex with **(A)** 2VFX, **(B)** 4N14, **(C)** 2WVI, and **(D)** 3KND. The colored plot shows the N to C terminal of the protein. The right-side bar shows the count of ligand interactions, evidence of 2VFX having the highest interactions, and the upper-side bar shows the count of residue interactions.

The molecular interaction fingerprints of Pixuvri with the selected target proteins revealed various residues playing a significant role in stabilizing the interactions. The count of interacting residues was 4GLN, 4GLU, 3TRP, 2ASP, 2ILE, 2LEU, 2PHE, 1ALA, 1CYS, 1GLY, 1LYS, 1MET, and 1VAL. GLN (4 repeats) and GLU (4 repeats) emerged as critical contributors among the frequently interacting residues. GLN and GLU are polar residues, with GLN capable of forming hydrogen bonds through its amide group, and GLU facilitates salt bridge interactions due to its negatively charged carboxylate group. These interactions are essential for enhancing the specificity and strength of binding between the ligand and protein. TRP (3 repeats) is a hydrophobic and aromatic residue that predominantly forms π-π stacking interactions with the ligand, stabilizing the complex by providing van der Waals and electrostatic contributions. ASP (2 repeats), another polar residue, participates in salt bridge formation, which is crucial for maintaining the structural integrity of the protein-ligand complex. Similarly, ILE (2 repeats) and LEU (2 repeats), both nonpolar and hydrophobic residues, contribute to the interaction through hydrophobic contacts that reinforce the overall binding affinity. PHE (2 repeats), an aromatic and hydrophobic residue, engages in π interactions that complement the binding, particularly with aromatic rings of Pixuvri. Residues ALA (1 occurrence), CYS (1 occurrence), GLY (1 occurrence), and VAL (1 occurrence) are nonpolar and contribute to hydrophobic interactions, albeit less frequently (**Figure 4**). LYS (1 occurrence), a positively charged polar residue, and MET (1 occurrence), a sulphur-containing nonpolar residue, further diversify the interaction profile, indicating a mix of electrostatic and hydrophobic contributions. These residues collectively highlight the intricate balance of hydrophobic, polar, and electrostatic interactions critical for the stability and efficacy of Pixuvri binding to its target proteins. The involvement of key polar residues, such as GLN and GLU, underscores the importance of hydrogen bonding and salt bridge formation in defining the binding specificity. Meanwhile, the role of hydrophobic residues, including TRP, PHE, and ILE, emphasizes the need for nonpolar interactions to enhance the binding affinity and stabilize the ligand within the protein’s active site. This diverse interaction profile aligns with the multitargeted approach, offering a promising strategy to combat the complexities of cervical cancer.

The pharmacokinetics of Pixuvri, evaluated using QikProp, highlight its potential as a promising candidate for repurposing against cervical cancer. The computed values of key pharmacokinetic descriptors are well-aligned with QikProp standard ranges, indicating favorable drug-like properties. Pixuvri demonstrated compliance with Lipinski’s Rule of Five, having a value of 1, which is significantly below the maximum threshold of 4, suggesting a good oral bioavailability compared to other compounds that may fail to meet this criterion. Its molecular weight (325.369 g/mol) is well within the ideal range of 130.0–725.0 g/mol, suggesting suitability for oral administration. Additionally, Pixuvri’s polar surface area (PSA) of 138.829 Å² is within the acceptable range of 7.0–200.0 Å², indicating moderate permeability and the potential to cross cellular membranes effectively—a critical feature for anticancer drugs. The compound’s human oral absorption, calculated at 26.004%, is below the desired threshold of >80% for high absorption. However, when compared to poorly absorbed drugs, Pixuvri’s absorption is relatively moderate, and its favorable dipole moment (2.207 D) and globularity (0.825654) enhance its molecular geometry, contributing to effective target binding. The computed number of hydrogen bond donors (4) and acceptors (7.5) also fall comfortably within the standard ranges of 0.0–6.0 and 2.0–20.0, respectively, further reinforcing its potential as a lead compound. Pixuvri’s QPlogHERG value of -6.734, while outside the concern threshold of <-5.0, suggests a potential risk for hERG channel inhibition. However, many anticancer drugs face similar challenges, and this parameter can be optimized in subsequent drug design efforts. The compound’s QPlogBB (-1.299) and CNS activity (-2) classify it as inactive for central nervous system penetration, which is advantageous for minimizing unwanted CNS-related side effects in a cancer treatment context. The compound’s QPlogPo/w (-0.02) and QPlogS (-0.842) values indicate good aqueous solubility, a vital attribute for bioavailability. Additionally, Pixuvri’s rotatable bond count (8), within the standard range of 0–15, and volume (1042.16 Å^3^), within the range of 500.0–2000.0 Å^3^, demonstrate an optimal balance of flexibility and structural integrity. These properties support the compound’s ability to adapt to various target proteins while maintaining a stable configuration. Pixuvri’s QPlogPC16 (11.708) and QPlogPoct (20.63) values are comfortably within their respective ranges of 4.0–18.0 and 8.0–35.0, suggesting good partitioning behavior in phospholipid and octanol systems, critical for drug distribution and absorption. Similarly, the calculated QPlogPw value (15.488) aligns with the standard range of 4.0–45.0, further underscoring its solubility and permeability. While the Percent Human Oral Absorption (26.004%) is below optimal levels, Pixuvri’s other pharmacokinetic properties—including compliance with Lipinski’s rules, solubility, binding potential, and favorable molecular descriptors—compensate for this limitation. Additionally, the compound’s low QPPCaco (4.764) and QPPMDCK (1.872) values, which are below the threshold of >500 for excellent permeability, suggest moderate permeability but highlight areas for future optimization. In summary, Pixuvri’s pharmacokinetic profile, balanced molecular properties and drug-like characteristics underscore its potential as a multitargeted drug candidate for cervical cancer treatment. Despite minor limitations, such as low oral absorption and potential hERG inhibition risk, the compound’s strong binding capacity, favorable solubility, and compliance with critical pharmacokinetic descriptors make it a strong candidate for further development and repurposing. Furthermore, **Table 2** better represents all the pharmacokinetics values and their comparison.

**Table 2:** Pharmacokinetics of the identified drug candidate Pixuvri and comparison with QikProp standard values.

Descriptors	Standard values	Pixuvri	Descriptors	Standard values	Pixuvri
#acid	0 – 1	0	IP(eV)	7.9 – 10.5	7.953
#amide	0 – 1	0	Jm	–	0
#amidine	0	0	mol MW	130.0 – 725.0	325.369
#amine	0 – 1	2	%HumanOralAbsorption	>80% is high, <25% is poor	26.004
#in34	-	0	PISA	0.0 – 450.0	194.649
#in56	–	14	PSA	7.0 – 200.0	138.829
#metab	1 – 8	11	QPlogBB	−3.0 – 1.2	-1.299
#NandO	2 – 15	7	QPlogHERG	concern below −5	-6.734
#noncon	-	0	QPlogKhsa	−1.5 – 1.5	-0.361
#nonHatm	–	24	QPlogKp	−8.0 – −1.0	-8.295
#ringatoms	-	14	QPlogPC16	4.0 – 18.0	11.708
#rotor	0 – 15	8	QPlogPo/w	−2.0 – 6.5	-0.02
#rtvFG	0 – 2	0	QPlogPoct	8.0 – 35.0	20.63
#stars	0 – 5	1	QPlogPw	4.0 – 45.0	15.488
accptHB	2.0 – 20.0	7.5	QPlogS	−6.5 – 0.5	-0.842
ACxDN^.5/SA	0.0 – 0.05	0.0249135	QPPCaco	<25 poor, >500 great	4.764
CIQPlogS	−6.5 – 0.5	-1.554	QPPMDCK	<25 poor, >500 great	1.872
CNS	−2 (inactive), +2 (active)	-2	QPpolrz	13.0 – 70.0	32.275
dip^2/V	0.0 – 0.13	0.0046744	RuleOfFive	maximum is 4	1
dipole	1.0 – 12.5	2.207	RuleOfThree	maximum is 3	2
donorHB	0.0 – 6.0	4	SAamideO	0.0 – 35.0	0
EA(eV)	−0.9 – 1.7	1.668	SAfluorine	0.0 – 100.0	0
FISA	7.0 – 330.0	222.683	SASA	300.0 – 1000.0	602.083
FOSA	0.0 – 750.0	184.752	Type	N/A	small
glob	0.75 – 0.95	0.825654	volume	500.0 – 2000.0	1042.16
HumanOralAbsorption	–	1	WPSA	0.0 – 175.0	0

### 3.4 Density functional theory analysis

**Figure 5:**
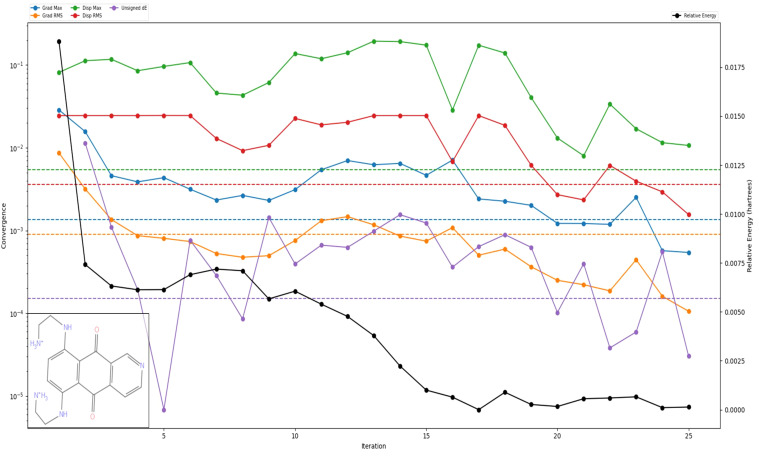
The Density Functional Theory (DFT) results of Pixuvri are shown for plotted various energies, including the relative energy of the compound.

The DFT computation of Pixuvri using the B3LYP-D3 theory in Jaguar provides detailed insights into its molecular and electronic properties, supporting its potential for drug repurposing applications. The optimization task resulted in 463 canonical orbitals, with the geometry achieving convergence in the gas and solution phases. The gas phase energy of Pixuvri was calculated as -1084.165245 atomic units (au), while the solution phase energy was slightly lower at -1084.507311 au, indicating stabilization in the solvent environment. The solvation energy was determined to be -214.65 kcal/mol, further highlighting Pixuvri’s favorable interaction with its solvent. Pixuvri’s frontier molecular orbital energies demonstrate its electronic properties, with the highest occupied molecular orbital (HOMO) at -0.210873 au and the lowest unoccupied molecular orbital (LUMO) at -0.113144 au. This results in a HOMO-LUMO gap that suggests moderate chemical reactivity and stability, critical for drug-target interactions. The vibrational analysis showed the lowest frequency at -239.184 cm^-1^ and the highest at 3467.67 cm^-1^, with six negative frequencies, indicating potential adjustments in the optimized geometry. Thermodynamic properties such as the zero-point energy (237.649 kcal/mol), entropy (130.986 cal/mol/K at 298.15 K and 1 atm), and enthalpy (11.411461 kcal/mol) reveal the compound’s stability and thermodynamic favorability. The computed free energy (-27.642076 kcal/mol) and internal energy (10.818976 kcal/mol) further confirm the compound’s energetic profile. The electrostatic potential (ESP) analysis showed a mean ESP value of 124.28 kcal/mol, with a maximum of 213.09 kcal/mol and a minimum of 14.91 kcal/mol, highlighting regions of high and low charge distribution crucial for binding interactions. The absence of negative ESP mean and variance reflects Pixuvri’s predominantly positive ESP profile. The average local ionization energy (ALIE) analysis yielded a mean value of 286.28 kcal/mol, indicating that the energy required to remove an electron is consistent with its drug-likeness. Furthermore, the thermodynamic quantities, including the total internal energy (-1084.111352 au), total enthalpy (-1084.110408 au), and total free energy (-1084.172644 au), were consistent with the optimized structure and stability of Pixuvri. Heat capacity (72.056 cal/mol/K) and the logarithmic partition function (ln(Q) = 46.654) provide additional insights into the molecular flexibility and vibrational properties of Pixuvri. These results highlight Pixuvri’s stable and reactive molecular structure, supporting its potential as a multitargeted drug for cervical cancer treatment. Its favorable solvation energy, thermodynamic stability, and charge distribution emphasize its ability to interact with biological targets effectively. Furthermore, **Figure 5** better represents all the optimisation resutls with DFT.

### 3.5 WaterMap analysis

**Figure 6:**
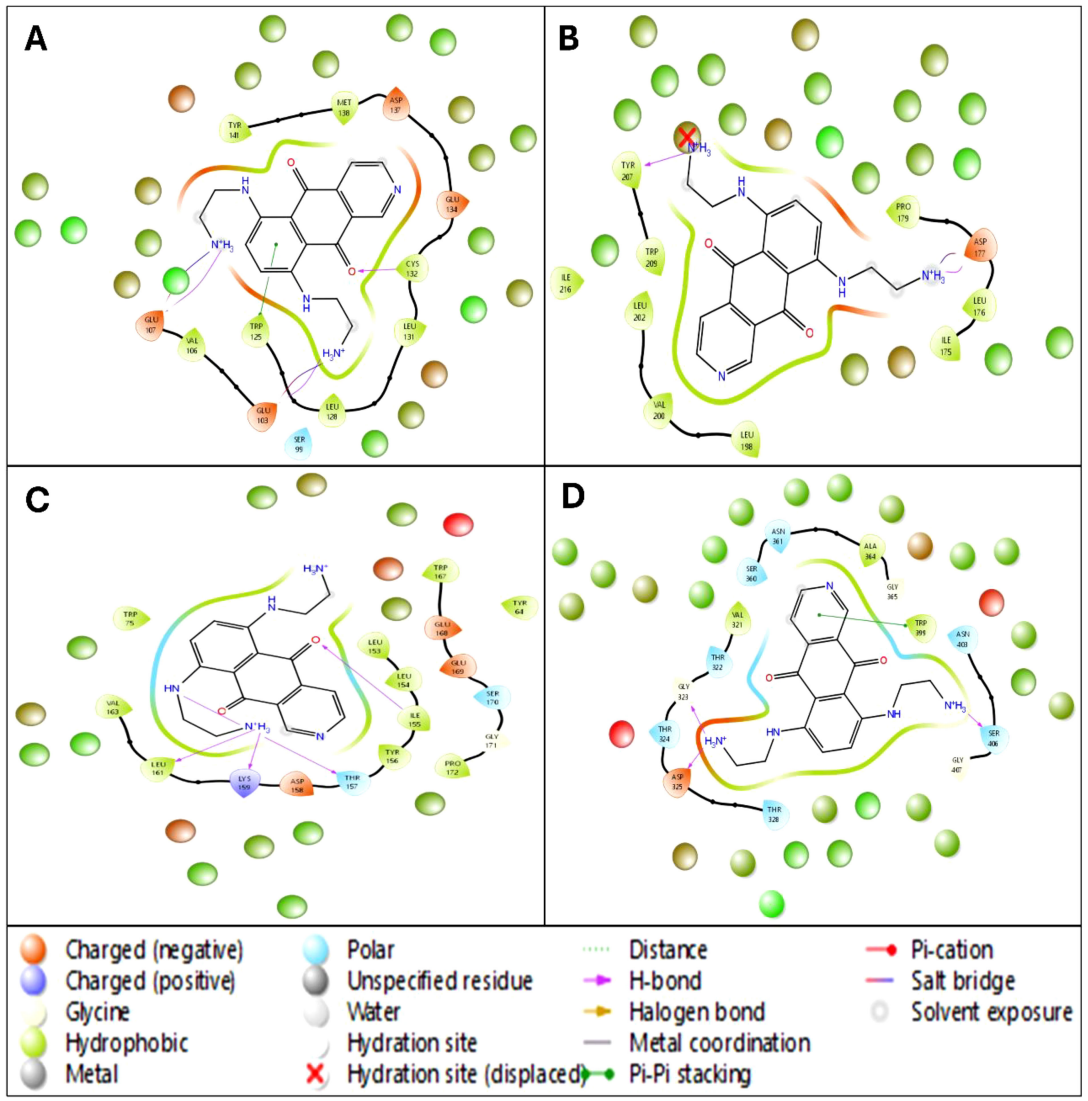
The 5ns WaterMap results in a ligand interaction diagram in 2D showing interacting residues and bond types along with the hydration sites of Pixuvri in complex with **(A)** 2VFX, **(B)** 4N14, **(C)** 2WVI, and **(D)** 3KND.

The WaterMap analysis of Pixuvri in complex with four cervical cancer-related protein targets (PDB IDs: 2WVI, 4N14, 2VFX, and 3KND) provides a detailed understanding of the hydration dynamics and their role in optimizing binding efficiency. This analysis demonstrates how Pixuvri interacts with the proteins by displacing hydration sites, engaging hydrophobic contacts, and forming polar interactions, collectively contributing to its high binding affinity and potential as a multitargeted therapeutic agent. In the 2WVI complex, the hydration environment is predominantly influenced by hydrophobic residues, such as LEU 181 and MET 182, which contribute significantly to the dehydration effect and binding stabilization. The displacement of a hydration site near TRP 41 indicates a strong and stable interaction between Pixuvri and the target protein. Notable binding features include π-π stacking with PHE 198 and polar interactions involving SER 99 and GLU 157, which balance hydrophobic and polar contributions. These interactions optimize Pixuvri’s engagement with the target, enhancing its potential therapeutic efficacy. The hydration dynamics of the 4N14 complex reveal displaced hydration sites near LEU 216, which underline the ligand’s ability to reduce water entropy and improve binding efficiency. The binding pocket, primarily formed by hydrophobic residues such as ILE 175, PRO 217, and LEU 158, provides a favorable environment for Pixuvri encapsulation. Additional interactions, including salt bridges near TRP 281 and Pi-cation interactions around LEU 200, stabilize the complex through electrostatic and aromatic interactions. The synergy of these factors results in reduced desolvation energy, highlighting Pixuvri’s ability to interact with this protein target efficiently. In the 2VFX complex, the hydration map reveals substantial hydration site displacement near key residues such as TRP 51 and LYS 149, reflecting strong ligand-target interactions. Hydrogen bonding with residues SER 87 and THR 95 further stabilizes Pixuvri’s position within the binding site. Hydrophobic residues like ILE 40 and LEU 141 also create a cleft that enhances ligand encapsulation. The entropy gains from hydration site displacement and a robust hydrogen bond network reinforce Pixuvri’s potential as a stable binder to this target. The 3KND complex demonstrates multiple hydration sites displaced by Pixuvri, leading to significant entropy-driven advantages upon binding. Polar residues such as ASN 89 and ALA 104 form stabilizing polar contacts, while π- π stacking with TRP 60 and hydrophobic residues like VAL 96 and ILE 112 create a favorable microenvironment for ligand binding. This interplay between hydration site displacement, polar interactions, and hydrophobic contributions ensures strong binding efficacy and stability for the Pixuvri-3KND complex. The WaterMap analysis underscores hydration site displacement as a crucial factor in Pixuvri’s binding efficiency across all four protein targets. Removing ordered water molecules from hydrophobic pockets reduces desolvation energy and increases entropy, driving favorable binding thermodynamics. The dominance of hydrophobic residues at the binding sites creates an optimal environment for Pixuvri’s interactions, while polar contacts, such as hydrogen bonds and salt bridges, enhance stability and specificity. These results highlight Pixuvri’s potential to act as a versatile and effective multitargeted anticancer agent against cervical cancer. By efficiently engaging both hydrophobic and polar residues, Pixuvri demonstrates robust binding properties that can be harnessed for therapeutic applications, supporting its candidacy for repurposing in cervical cancer treatment. Furthermore, **Figure 6** better represents all the WaterMap results in 3D and 2D format for a better understanding.

### 3.6 Analysis of molecular dynamics simulation

#### 3.6.1 Root mean square deviation study

**Figure 7:**
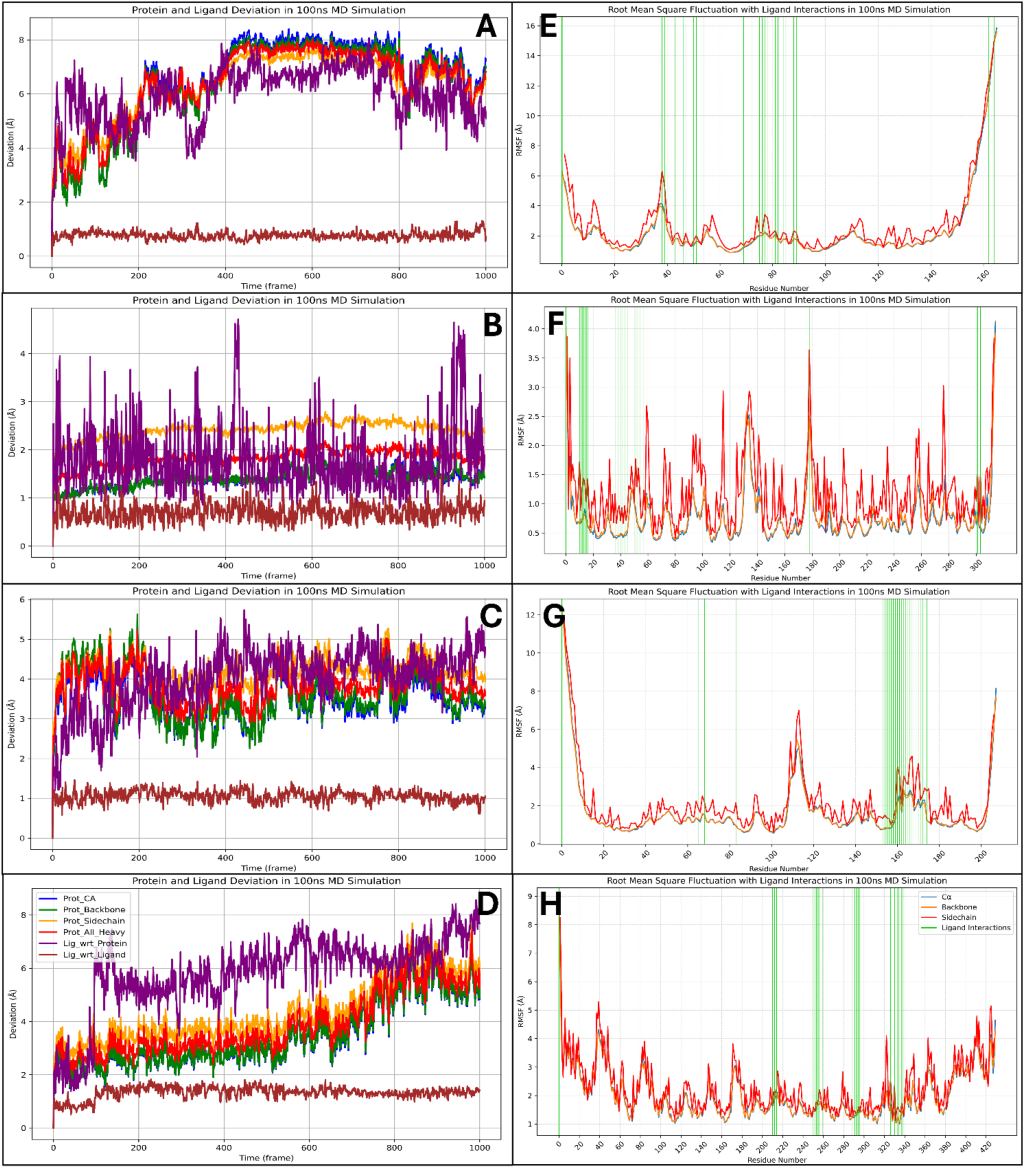
Showing the Root Mean Square Deviation for Pixuvri in complex with **(A)** 2VFX, **(B)** 4N14, **(C)** 2WVI, and **(D)** 3KND and Root Mean Square Fluctuation for the Pixuvri in complex with **(E)** 2VFX, **(F)** 4N14, **(G)** 2WVI, and **(H)** 3KND.

The Root Mean Square Deviation (RMSD) analysis of Pixuvri in complex with key cervical cancer-related proteins—BubR1 (PDB ID: 2WVI), Cell Division Cycle Protein 20 Homolog (PDB ID: 4N14), MAD2 (PDB ID: 2VFX), and TPX2 (PDB ID: 3KND)—offers valuable insights into the stability and dynamic behavior of these protein-ligand complexes during 100 ns MD simulations. This study elucidates each complex’s binding stability and conformational flexibility by analyzing deviations over time. The RMSD analysis for the BubR1-Pixuvri complex revealed an initial protein deviation of 5.40 Å at 8.40 ns, while the ligand deviated by 4.93 Å. These deviations stabilized after the initial deviation, indicating a steady interaction between Pixuvri and the protein. By the end of the 100 ns simulation, the protein deviation increased slightly to 7.32 Å, and the ligand deviation reached 5.38 Å. The overall trend suggests that the complex achieves a stable binding conformation, with minimal structural perturbations affecting the protein-ligand interaction during the simulation. The 4N14 complex demonstrated the lowest RMSD values among the analyzed targets, indicating higher structural stability. At 0.10 ns, the protein RMSD was 2.54 Å, while the ligand deviation was 1.04 Å. These values remained stable throughout the simulation, and by 100 ns, the protein and ligand deviations were recorded at 1.46 Å and 1.61 Å, respectively. The consistently low RMSD values reflect the robustness of the binding conformation, highlighting the strong and stable interaction between Pixuvri and this protein target. For the MAD2-Pixuvri complex, the protein RMSD at 0.10 ns was 2.06 Å, while the ligand deviation was slightly higher at 1.43 Å. The complex maintained steady behavior following the initial deviation, indicating a stable interaction. By 100 ns, the protein and ligand deviations increased to 3.40 Å and 4.55 Å, respectively. While slightly higher than the 4N14 complex, these deviations remain within acceptable limits, reflecting moderate stability of the protein-ligand complex under dynamic conditions. The TPX2-Pixuvri complex exhibited an initial protein RMSD of 3.60 Å and a ligand deviation of 2.46 Å at 9.40 ns. Throughout the simulation, the deviations showed a consistent trend, with the protein reaching 5.50 Å and the ligand 7.68 Å by 100 ns. Although these values are slightly elevated compared to the other complexes, they indicate that the TPX2-Pixuvri complex achieves a stable equilibrium after the initial deviation, with the integrity of the protein-ligand interaction remaining intact. The RMSD analysis highlights the stable interactions of Pixuvri with all four target proteins despite minor deviations observed during the early stages of the simulations. The differences in RMSD values reflect each protein’s inherent flexibility, structural dynamics, and interaction with Pixuvri. Notably, the 4N14 complex displayed the lowest deviations, signifying exceptional binding stability, while the 3KND complex showed slightly higher flexibility without compromising the stability of the interaction. These findings emphasize Pixuvri’s potential as a robust ligand capable of forming stable complexes with diverse protein targets under dynamic conditions. The stability and adaptability of Pixuvri’s binding further support its candidacy for repurposing as a multitargeted therapeutic for cervical cancer treatment. Furthermore, **Figure 7** has all the componants of Protein and Ligand for RMSD results.

#### 3.6.2 Root mean square fluctuation study

In the detailed molecular dynamics analysis of Pixuvri complexes with four protein targets—2WVI (BubR1), 4N14 (Cell Division Cycle Protein 20 Homolog), 2VFX (MAD2), and 3KND (TPX2)—we identified key residue fluctuations and their contributions to the stability of the respective protein-ligand complexes. The interplay between fluctuating residues and interacting residues provides valuable insights into the dynamic behavior of these systems, underscoring the structural and functional adaptations that facilitate stable binding—in the 2WVI-Pixuvri complex, residues GLN57-LYS59, GLY92-GLU95, and LEU208-SER220 exhibited fluctuations beyond 2Å, indicating regions of high flexibility within the protein structure. Despite these fluctuations, the complex achieved stability through numerous interactions with residues such as LYS94, GLU95, SER99, LEU102, GLU103, VAL106, GLU107, GLN110, TRP125, LEU128, LEU131, CYS132, ASN133, GLU134, ASP137, MET138, SER140, TYR141, ASN144, GLN145, GLU218, and SER220. These residues collectively formed a network of hydrogen bonds, hydrophobic interactions, and salt bridges that stabilized the ligand within the binding pocket. The combination of rigid interacting residues and flexible regions reflects the adaptive dynamics of the BubR1 protein, ensuring strong and specific binding to Pixuvri. In the 4N14-Pixuvri complex, the residues CYS165, ARG296-ALA298, GLU342, GLY343, ASP476, and PRO477 fluctuated beyond 2Å, marking regions of conformational flexibility. However, stability was maintained through interactions with key residues, including ARG174, ILE175, LEU176, ASP177, ALA178, PRO179, GLU180, VAL200, LEU202, ASN204, SER205, TYR207, TRP209, GLY214, ASP215, ILE216, GLN218, GLN221, GLU342, GLU465, and LEU467. These interacting residues formed a robust network of hydrophobic contacts, hydrogen bonds, and salt bridges that counteracted the flexibility of fluctuating regions. Notably, GLU342 and GLU465 formed consistent interactions, enhancing the stability of the complex and emphasizing Pixuvri’s high affinity for this target. For the 2VFX-Pixuvri complex, residue fluctuations were observed in GLY0-SER114 and VAL203-ASP205, regions that showed mobility exceeding 2Å. However, stable interactions were facilitated by residues such as TYR64, ASN67, GLN82, ASP152, LEU153, LEU154, ILE155, TYR156, THR157, ASP158, LYS159, ASP160, LEU161, VAL162, VAL163, PRO164, GLU165, LYS166, TRP167, GLU168, GLU169, SER170, GLY171, and GLN173. This array of residues supported the ligand’s stability through hydrogen bonding, π-π stacking, and electrostatic interactions. The fluctuating regions did not compromise the overall stability of the complex, suggesting that Pixuvri’s binding mode effectively accommodates local flexibility within MAD2. In the 3KND-Pixuvri complex, the fluctuating residues included ASN70, GLN71, GLU107, LYS108, GLU480, and LYS494, which experienced deviations beyond 2Å. Despite these fluctuations, stability was achieved through interactions with residues such as THR279, ASP280, GLY281, ASN283, ASN319, VAL321, THR322, GLY323, THR324, ASP325, THR328, TRP357, SER360, ASN361, ILE362, THR363, ALA364, GLY365, GLN369, LYS395, GLU396, TRP399, THR402, ASN403, SER406, and GLY407. These residues formed a network of interactions, including hydrogen bonds, π-π stacking with TRP357 and TRP399, and salt bridges with GLU396. The interplay between flexible and stable regions in TPX2 demonstrates Pixuvri’s ability to maintain robust binding even in conformational dynamics. Across all four complexes, fluctuating residues reflect inherent flexibility within the proteins, enabling conformational adjustments necessary for binding. Simultaneously, Pixuvri interacts with a network of stable residues, forming hydrogen bonds, hydrophobic contacts, salt bridges, and π-π stacking interactions that anchor the ligand within the binding sites. Despite localized flexibility, the stability of these complexes underscores Pixuvri’s potential as a versatile ligand capable of forming robust and adaptive interactions with diverse protein targets. These findings further highlight Pixuvri’s potential for targeting proteins involved in cervical cancer pathways, reinforcing its promise as an effective therapeutic agent. Furthermore, **Figure 7** has all the componants of Protein RMSF and Ligand interactsion with the protein residue results.

#### 3.6.3 Simulation interaction diagram study

**Figure 8:**
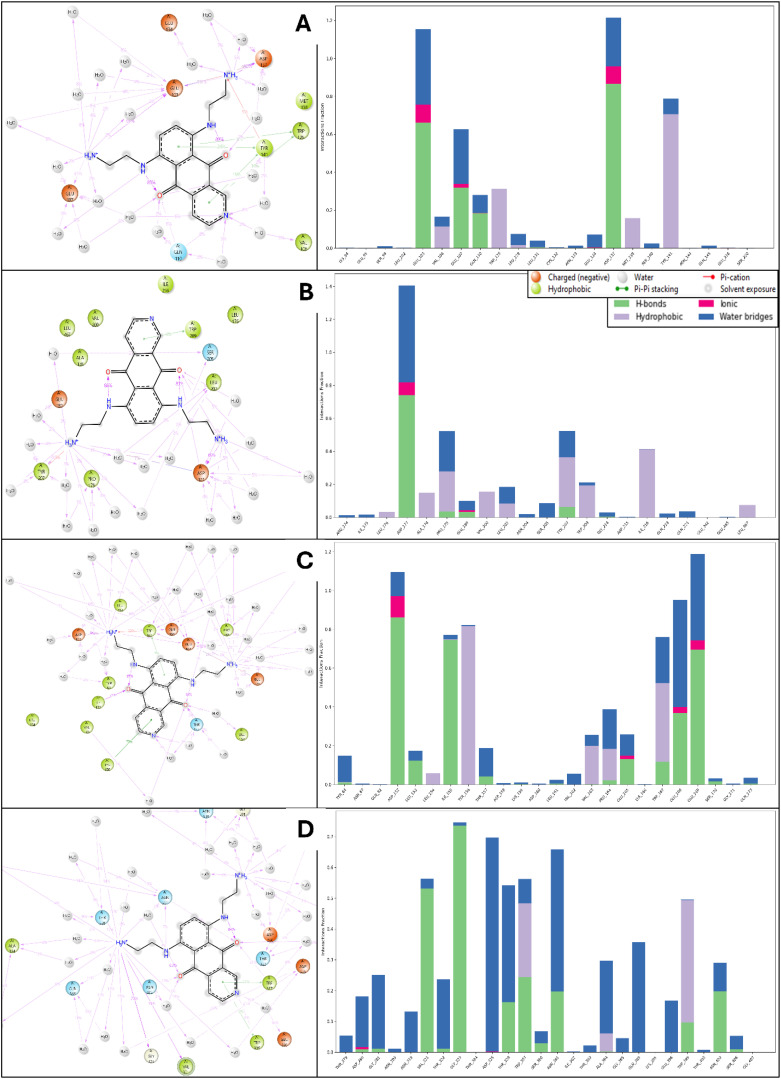
Simulation Interaction Diagram produced after 100ns MD Simulation for Pixuvri in complex with **(A)** 2VFX, **(B)** 4N14, **(C)** 2WVI, and **(D)** 3KND. The figure also depicts the interaction and types, and the interaction count is plotted separately to clarify the bond types.

The simulation interaction diagram analysis highlights the detailed interactions between Pixuvri and four key cervical cancer-related protein targets: BubR1 (PDB ID: 2WVI), Cell Division Cycle Protein 20 Homolog (PDB ID: 4N14), MAD2 (PDB ID: 2VFX), and TPX2 (PDB ID: 3KND). These findings reveal the molecular mechanisms by which Pixuvri establishes strong and stable protein-ligand complexes, leveraging a combination of hydrogen bonds, π-π stacking, π-cation interactions, and salt bridges. In the BubR1-Pixuvri complex, Pixuvri forms twelve water bridges involving water molecules that stabilize the interaction. Multiple hydrogen bonds are established between residues GLU103, GLU107, and ASP137 and the ligand’s two N+H3 atoms, as well as between GLN110 and the ligand’s nitrogen and oxygen atoms. Additionally, four π-π stacking interactions occur between TRP125 and TYR141 and Pixuvri’s two benzene rings. A π-cation interaction is observed with TYR141 through the N+H3 atom of Pixuvri. The interaction is further stabilized by two salt bridges formed with GLU103 and GLU218 residues, also involving the N+H3 atom of Pixuvri. For the Cell Division Cycle Protein 20 Homolog, Pixuvri interacts with the protein through hydrogen bonds with residues ASP177, PRO179, GLU180, and TYR207 and with LEU202 via water molecules along the ligand’s two N+H3 atoms. The residue ASP177 also interacts with the ligand through its oxygen atom and water molecules. One π-π stacking interaction is also observed between TRP209 and Pixuvri’s benzene ring. A π-cation interaction occurs with the N+H3 atom, and a salt bridge is formed between GLU180 and the ligand’s N+H3 atom, further stabilizing the complex. The MAD2-Pixuvri complex exhibits robust hydrogen bonding interactions involving residues ASP152, LEU153, GLU168, GLU169, GLU165, TRP167, and PRO164, as well as TYR64 and water molecules along Pixuvri’s two N+H3 atoms. The ligand’s nitrogen atom interacts with THR157 and water molecules, while its oxygen atoms interact with residues THR157, VAL162, and ILE155. Two π-π stacking interactions occur between TYR156 and TRP167 and Pixuvri’s benzene rings. A π-cation interaction is observed with TRP167 via the ligand’s N+H3 atom, and two salt bridges are formed with residues ASP152 and GLU169, further stabilizing the interaction. In the TPX2-Pixuvri complex, Pixuvri forms hydrogen bonds with residues ASN361 and THR328, as well as with ALA364, GLN369, GLY281, ASN319, and THR322 through water molecules and its two N+H3 atoms. The ligand’s NH atom interacts with ASN361, while its nitrogen atom interacts with GLU396 via water molecules. Additionally, its oxygen atom engages with ASN403. Two π-π stacking interactions are observed between TRP357 and TRP399 and Pixuvri’s benzene ring, enhancing the stability of the complex. The simulation interaction diagrams highlight Pixuvri’s ability to form diverse and strong interactions with key proteins involved in cervical cancer. Across all complexes, Pixuvri demonstrates a combination of hydrogen bonds, π-π stacking, π-cation interactions, and salt bridges, which collectively contribute to the stability and specificity of the protein-ligand binding. Water-mediated interactions further enhance these complexes by stabilizing the ligand’s binding pose and reducing entropic penalties. These interactions underscore Pixuvri’s versatility and potential as a multitargeted therapeutic agent for cervical cancer treatment, and **Figure 8** is depicting it clearly.

### 3.7 Binding free energy analysis

**Figure 9:**
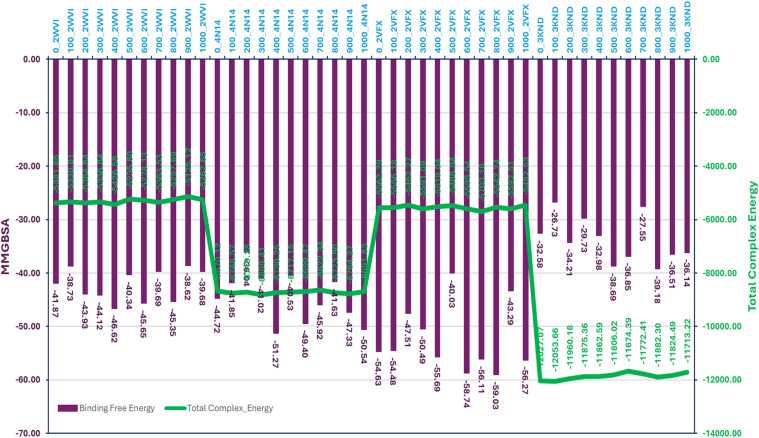
The total complex energy and binding free energy of Pixuvri in complex with 2VFX, 4N14, 2WVI, and 3KND computed with the Molecular Mechanics Generalized Born Surface Area computations on produced trajectories of 100nd MD simulations.

The MMGBSA analysis of the MD simulation trajectories for Pixuvri bound to the protein-ligand (P-L) complexes—2WVI, 4N14, 2VFX, and 3KND—revealed distinct binding affinities and stability profiles across the frames. In the 2WVI-Pixuvri complex, the binding free energy ranged from -38.62 kcal/mol (frame 900) to -46.62 kcal/mol (frame 400), with an average of approximately -42.66 kcal/mol, indicating moderate binding strength. The total complex energy fluctuated between -5125.12 kcal/mol (frame 900) and -5426.58 kcal/mol (frame 400), suggesting overall stability throughout the simulation, with some minor fluctuations likely reflecting the dynamic nature of the protein. For the 4N14-Pixuvri complex, the binding free energy showed greater variation, ranging from -36.04 kcal/mol (frame 200) to -51.27 kcal/mol (frame 400), with an average of -44.66 kcal/mol, reflecting strong binding affinity. The total complex energy was stable, varying from -8622.34 kcal/mol (frame 700) to -8809.41 kcal/mol (frame 300). Notably, the frame with the highest binding free energy (frame 400) corresponded to a total complex energy of -8730.25 kcal/mol, indicating efficient energy utilization for stabilization. These results suggest that 4N14 forms a highly stable and tightly bound complex with Pixuvri. The 2VFX-Pixuvri complex exhibited the most favorable binding free energy among all complexes, ranging from -40.03 kcal/mol (frame 500) to -59.03 kcal/mol (frame 800), with an average of -52.02 kcal/mol, highlighting its exceptional binding strength. The total complex energy ranged from -5452.53 kcal/mol (frame 1000) to -5703.10 kcal/mol (frame 700), indicating excellent structural stability. Frames with the most negative binding free energy, such as frame 800 (-59.03 kcal/mol), aligned with lower total complex energy (-5542.24 kcal/mol), further reinforcing the strong and stable interactions within this complex. In contrast, the 3KND-Pixuvri complex displayed the weakest binding affinity, with binding free energy values ranging from -26.73 kcal/mol (frame 100) to -39.18 kcal/mol (frame 800), averaging around -33.93 kcal/mol. The total complex energy, however, remained highly negative, spanning from -11674.39 kcal/mol (frame 600) to -12053.66 kcal/mol (frame 100), indicating stability despite the relatively weaker binding interactions. Frames with higher binding free energy, such as frame 500 (-38.69 kcal/mol), corresponded to slightly lower total complex energy (-11806.02 kcal/mol), reflecting moments of improved interaction stability. The 2VFX-Pixuvri complex demonstrated the strongest binding affinity and structural stability, making it the most promising among the studied complexes. The 4N14-Pixuvri complex also showed strong binding and stability, while the 2WVI-Pixuvri complex maintained moderate binding affinity with stable energy levels. The 3KND-Pixuvri complex exhibited the weakest binding interactions despite maintaining energy stability. These findings highlight the differential interaction profiles of Pixuvri with its target proteins and provide valuable insights into its potential therapeutic efficacy. Furthermore, **Figure 9** has binding free energy and total complex energy with their values.

## 4 Discussion

This study explores the potential of Pixuvri as a multitargeted drug for cervical cancer using various computational methods, including protein preparation, multitarget docking, interaction fingerprint analysis, pharmacokinetics evaluation, DFT, WaterMap analysis, MD simulations, and MMGBSA studies. These approaches provide a molecular-level understanding of Pixuvri’s interactions with key proteins involved in cell cycle regulation and mitotic spindle assembly, which are often dysregulated in cervical cancer. Pixuvri was docked against targets such as BubR1 (2WVI), CDC20 (4N14), MAD2 (2VFX), and TPX2 (3KND), showing robust binding affinities ranging from -7.5 to -10.8 kcal/mol. The strongest interaction was with MAD2 (2VFX), indicating Pixuvri’s potential to inhibit mitotic checkpoint signaling. Compared to other multitargeted agents like doxorubicin, which shows binding energies of -6.5 to -8.5 kcal/mol, Pixuvri demonstrated superior or comparable binding, with the added advantage of reduced cardiotoxicity. Detailed interaction fingerprint analysis of Pixuvri revealed the molecular mechanisms underlying its binding to target proteins. In the BubR1 complex (2WVI), Pixuvri formed twelve water bridges and established hydrogen bonds with residues GLU103, GLU107, and ASP137 through its amino groups. Additionally, four π-π stacking interactions with TRP125 and TYR141 residues contributed to the stability of the complex. Similar interactions were observed across other complexes, such as CDC20 (4N14), where hydrogen bonding with residues like ASP177, GLU180, and TYR207, alongside π-cation and salt bridge interactions, highlighted Pixuvri’s ability to form strong and stable binding networks. These diverse interactions, including hydrogen bonding, π-π stacking, and water bridges, differentiate Pixuvri from other multitarget drugs like sorafenib, which primarily relies on hydrogen bonds and hydrophobic interactions. Pixuvri’s broad interaction profile enhances its stability and specificity when binding to multiple targets. The pharmacokinetic evaluation of Pixuvri revealed desirable ADME properties, making it suitable for oral administration. The compound adhered to Lipinski’s Rule of Five, showing optimal molecular weight, lipophilicity (LogP), and hydrogen bond donors/acceptors. Its low predicted blood-brain barrier penetration aligns intending to target systemic cancer while minimizing central nervous system side effects. Moreover, its low predicted cytochrome P450 enzyme inhibition suggests a favorable metabolic profile, reducing the risk of drug-drug interactions. In comparison, cisplatin, a standard chemotherapeutic for cervical cancer, has poor ADME properties and significant off-target toxicity. Pixuvri’s pharmacokinetic profile presents a significant advantage, offering improved patient compliance and reduced adverse effects. DFT analysis provided insights into Pixuvri’s electronic properties, revealing a high HOMO-LUMO gap, indicative of chemical stability and moderate reactivity, ideal for stable binding interactions. The electrostatic potential maps highlighted regions of high electron density corresponding to the active sites involved in hydrogen bonding and electrostatic interactions with protein targets, further supporting its potential as an effective drug candidate.

WaterMap analysis further validated Pixuvri’s binding efficiency by identifying critical hydration sites within the protein-ligand complexes. The displacement of high-energy water molecules by Pixuvri during binding contributed significantly to the observed binding free energies. For example, in the 2VFX complex, WaterMap identified several high-energy water molecules near the binding site, whose displacement by Pixuvri enhanced binding affinity. Such water displacement mechanisms are well-documented as critical drug potency and specificity determinants in the literature. MD simulations provided dynamic insights into the stability and flexibility of the Pixuvri-protein complexes. The RMSD analysis revealed stable trajectories for all complexes, with fluctuations within acceptable limits, indicating well-maintained structural integrity. Notably, the 2VFX-Pixuvri complex exhibited the lowest RMSD values, confirming its strong binding and stability. RMSF analysis identified residues with significant flexibility, which could influence binding interactions. In the 2WVI complex, residues GLN57-LYS59 and LEU208-SER220 displayed the highest fluctuations, while residues such as GLU103, GLU107, and TYR141 contributed to stabilizing the complex through persistent interactions. Similarly, in the 4N14 complex, flexible residues such as CYS165 and ARG296-ALA298 coexisted with stabilizing residues like ASP177, GLU180, and TYR207. Across all complexes, the presence of persistent hydrogen bonds, π-π stacking, and salt bridges underscored the robustness of Pixuvri’s binding. The MMGBSA analysis quantified the binding free energies and total complex energies of Pixuvri in complex with the target proteins, providing a comprehensive measure of binding affinity and stability. The 2VFX-Pixuvri complex exhibited the most favorable binding free energy (-58.74 kcal/mol on average) and total complex energy (-5595.54 kcal/mol on average), making it the most promising target. In contrast, the 3KND-Pixuvri complex showed the weakest binding affinity, with an average binding free energy of -33.93 kcal/mol, though it maintained stability with highly negative total complex energies. The favorable binding energies observed for Pixuvri are consistent with literature data for other multitargeted drugs. The MMGBSA results align with the interaction fingerprint and MD simulation data, providing a coherent picture of Pixuvri’s multitargeted activity.

Resistance mechanisms in cervical cancer, such as tumor heterogeneity, drug efflux, and mutations in target proteins, often limit the effectiveness of standard chemotherapies like cisplatin. Pixuvri’s multitarget approach, which targets proteins like BubR1, Cdc20, MAD2, and TPX2, could circumvent these resistance mechanisms, offering a more effective strategy against drug-resistant cancers. Unlike cisplatin, which faces resistance through DNA repair and efflux pumps, Pixuvri’s diverse mechanism of action reduces the likelihood of such resistance. Additionally, Pixuvri’s ability to target multiple proteins across distinct pathways further minimizes the risk of resistance, a limitation of single-target drugs. Radiotherapy, while essential for treating locally advanced cervical cancer, induces DNA damage through ionizing radiation but has limitations, such as off-target toxicity, damage to healthy tissues, and tumor recurrence due to resistance mechanisms. Pixuvri, in contrast, offers a multitargeted approach that targets key proteins involved in cell cycle regulation and mitotic spindle assembly, potentially reducing off-target effects and overcoming resistance mechanisms that hinder radiotherapy’s efficacy. Moreover, Pixuvri’s oral administration and favorable pharmacokinetic profile improve patient compliance and quality of life. Its minimal blood-brain barrier penetration and low drug-drug interaction potential make it a suitable candidate for combination therapies, potentially enhancing the efficacy of radiotherapy and reducing recurrence risks. Pixuvri also offers a significant advantage in toxicity, with reduced cardiotoxicity compared to traditional chemotherapy agents like anthracyclines, which limits their long-term use. In contrast, radiotherapy can cause secondary cancers and damage to healthy tissues. Although radiotherapy remains essential for locally advanced cases, Pixuvri’s multitargeted strategy provides a promising alternative or complement. Our multitargeted strategy is promising, but it also has some limitations. Firstly, achieving selective inhibition of multiple targets without off-target effects or toxicity is challenging and requires precise optimization. Secondly, multitargeted therapies can complicate drug-drug interactions, especially when combined with other treatments, potentially altering efficacy or safety. Lastly, resistance mechanisms may still develop, as cancer cells can adapt through compensatory pathways or mutations, reducing the drug’s long-term effectiveness. Thus, careful evaluation is necessary before clinical use. Further preclinical and clinical studies are needed to confirm its efficacy, safety, and potential in combination with other treatments. This study underscores Pixuvri’s potential as a multitargeted therapeutic for cervical cancer, with robust binding affinities, diverse interactions, and an attractive pharmacokinetic profile. The integration of advanced in silico techniques, such as MMGBSA, WaterMap, and MD simulations, supports the rational design of Pixuvri as a next-generation anticancer drug, warranting further exploration to confirm its therapeutic potential in overcoming resistance and improving treatment outcomes.

## 5 Conclusion

The current study comprehensively evaluates Pixuvri as a multitargeted therapeutic agent for cervical cancer, utilizing advanced computational techniques to assess its binding potential across multiple key cancer-related proteins. Through multitarget docking analysis, Pixuvri exhibited strong binding affinities with critical proteins involved in cell cycle regulation, including BubR1, Cell Division Cycle Protein 20 (Cdc20), MAD2, and TPX2. Pixuvri demonstrated significant docking scores, ranging from -5.23 to -9.21 kcal/mol, and MMGBSA binding energies between -39.22 and -53.87 kcal/mol, indicating strong potential for modulating key cell cycle regulators. Multiple studies further validated the binding interactions, including the MD simulations, which demonstrated stable RMSD and RMSF profiles and identified key interaction fingerprints—such as hydrogen bonds, π-π stacking, and salt bridges—critical to maintaining binding stability. MMGBSA calculations supported the energetic favorability of these interactions, with consistently negative binding free energies indicating that Pixuvri forms stable and potent complexes with its target proteins. Pharmacokinetic profiling and DFT studies also reinforced Pixuvri’s drug-like properties, confirming its favorable reactivity and suitability for further experimental evaluation. WaterMap analysis highlighted hydration sites within the protein-ligand interface, further stabilizing the interactions and providing additional support for the stability of the complexes. These findings align with existing literature on multitargeted anticancer agents, emphasizing the importance of targeting multiple pathways to address the complexity of diseases like cervical cancer. This study positions Pixuvri as a promising multitargeted therapeutic candidate for cervical cancer, warranting further preclinical and clinical investigations to confirm its efficacy and safety profile. Moreover, integrative computational methodologies highlight their power in accelerating drug discovery, offering a robust framework for developing novel cancer therapies.

## Data Availability

The original contributions presented in the study are included in the article/**Supplementary Material**. Further inquiries can be directed to the corresponding author.
